# Machine Learning Models Using SHapley Additive exPlanation for Fire Risk Assessment Mode and Effects Analysis of Stadiums

**DOI:** 10.3390/s23042151

**Published:** 2023-02-14

**Authors:** Ying Lu, Xiaopeng Fan, Yi Zhang, Yong Wang, Xuepeng Jiang

**Affiliations:** 1 School of Resource and Environmental Engineering, Wuhan University of Science and Technology, Wuhan 430081, China; 2 Hubei Industrial Safety Engineering Technology Research Center, Wuhan 430081, China

**Keywords:** risk assessment model, stadium fire risk, equipment management, SHapley Additive exPlanations (SHAP), random forest algorithm

## Abstract

Machine learning methods can establish complex nonlinear relationships between input and response variables for stadium fire risk assessment. However, the output of machine learning models is considered very difficult due to their complex “black box” structure, which hinders their application in stadium fire risk assessment. The SHapley Additive exPlanations (SHAP) method makes a local approximation to the predictions of any regression or classification model so as to be faithful and interpretable, and assigns significant values (SHAP value) to each input variable for a given prediction. In this study, we designed an indicator attribute threshold interval to classify and quantify different fire risk category data, and then used a random forest model combined with SHAP strategy in order to establish a stadium fire risk assessment model. The main objective is to analyze the impact analysis of each risk characteristic on four different risk assessment models, so as to find the complex nonlinear relationship between risk characteristics and stadium fire risk. This helps managers to be able to make appropriate fire safety management and smart decisions before an incident occurs and in a targeted manner to reduce the incidence of fires. The experimental results show that the established interpretable random forest model provides 83% accuracy, 86% precision, and 85% recall for the stadium fire risk test dataset. The study also shows that the low level of data makes it difficult to identify the range of decision boundaries for Critical mode and Hazardous mode.

## 1. Introduction

With emerging information technology innovations [1] such as the Internet of Things (IoT) and Big Data, among others, machine learning is becoming increasingly important in assessing building fire risk and improving risk warning capabilities. Machine learning is relevant to various types of risk assessment, such as community-level building fire prediction (e.g., real estate [2], parking lots [3], public spaces, commercial complexes, and shopping centers [4]), property-level building fire assessment (using assessment metrics such as property damage [5], casualties [6], and incident severity [7,8]), and regional risk analysis [9]. Fire risk assessment of stadiums is one of the hot research topics in the field of fire safety. The tragic Bradford Stadium fire of 11 May 1985, which killed 56 people and seriously burned more than 200, and the fire accident at the Korea Sports Center on 21 December 2017, where the blaze killed 29 people and injured 29 others, caused great personal injuries and property damage in the fire accident. Once a fire accident occurs in a stadium, its fire hazards, evacuation and rescue and fire control difficulties, casualties, and losses are much greater than ordinary buildings. The manifestations of fire hazards in stadiums vary, but the root causes of fires in stadiums can be attributed to the following: (1) large building space, crowded, rapid fire spread, and difficult evacuation; (2) diverse functions and forms, combustible items, large fire loads; (3) new materials, smoke toxicity; (4) high space, conventional fire detectors, and fire extinguishing equipment cannot function effectively. It can be seen that once a fire occurs in a stadium, it will cause huge casualties and property damage, and the fire risk is high, so it is especially necessary to conduct a scientific and reasonable fire risk assessment.

Due to the complex and diverse risk factors of stadiums, the danger is high and the consequences of accidents are serious. The government and fire safety management departments mainly work in the mode of “human sea warfare to carry out inspections, turn-by-turn filtering focus” [10], and rely solely on human experience to determine whether the fire risk level is large, while the ability to proactively detect and advance warning is weak. In order to prevent and reduce the possibility of fire, the stadium fires need to be transferred from after-the-fact “fire rescue” to beforehand “assessment and prevention”, the early detection and prediction of potential fire hazards in the stadium and timely rectification. In many areas, the earliest decisions may be more valuable and support efficient decision-making. However, people may need more time to process information and draw conclusions. Since time is an important factor in risk decision-making, a small delay in the firefighting decision-making process in the event of a fire in a stadium could delay the first emergency response efforts, resulting in huge casualties and property damage. Therefore, the construction of an accurate and efficient fire risk assessment model for stadiums is an urgent scientific problem to be solved.

Using machine learning models, we can speed up the analysis of partial information and make it more objective and effective than human-based subjective analysis in terms of predictive performance. However, complex machine learning models are computationally expensive, and the obtained important input variables do not elaborate on the specific effects of the variables on the predicted individuals, the importance of the input variables in different categories in the classification problem, and the reasons why the input variables lead to a particular risk assessment, and the results are considered difficult to interpret. In addition, SHAP helps to explain various supervised learning models and assign significant values (SHAP value) to each input variable for a particular prediction. They use SHAP-based interpretable machine learning models to better capture the relationships between variables and between variables and specific targets. Although a large number of studies have explored the interpretability of machine learning models in the field of fire risk using game theory and local approximations, no relevant researchers have explored interpretable machine learning models for fire risk assessment in sports stadiums.

To further assist stadiums with fire supervision and management and resource planning, in the context of an IoT fire remote system platform, we use a real database of stadium fire risk and equipment management to obtain interpretable machine learning models. In this paper, an index attribute threshold interval is designed to classify and quantify different fire risk category data, and then a random forest model combined with SHAP strategy is used to establish a stadium fire risk assessment model to analyze the impact analysis of each risk feature on four different risk assessment models, so as to find the complex nonlinear relationship between risk features and stadium fire risk. In addition, risk assessment models for stadium fires are critical for (1) quickly assessing damage or failure of firefighting infrastructure equipment, (2) assessing whether the daily disposition of firefighting personnel management and hazard management is reasonable, and (3) finding key insights into the complex nonlinear behavior of risk characteristics and stadium fire risk. Its main purpose is to enable unit fire management to converge firefighting resources to rectify or eliminate major fire risk hazards in the first place, and to nip more fire hazards in the bud. In summary, the main contributions of this study are:

- We designed a risk prediction model based on random forest algorithm with SHapley Additive exPlanations strategy and developed a stadium fire risk assessment model. It can effectively identify and visually explain the importance and contribution of various fire risk factors to four different stadium fire risk assessment models.
- We designed an indicator attribute threshold interval to quantify and grade the fire risk assessment indicators.

The rest of this paper is organized as follows. Section 2 describes the work related to risk assessment and forecasting. Section 3 describes the framework and details of the interpretable machine learning approach, i.e., the stadium fire risk prediction method based on interpretable random forests. The application of the method to the stadium fire risk dataset and the equipment management dataset and the discussion of the interpretability analysis of the findings are presented in detail in Section 4. The limitations of this study are presented in Section 5. Finally, Section 6 discusses significant findings from the current study.

## 2. Related Work

Most of the existing work on fire risk assessment and prediction addresses forest land, forest fires and urban–forest boundaries, such as in China [11], Portugal [12], and Canada [13]. They use different methods such as random forests [11], general circulation models [13], and artificial neural networks [12], aiming to support the allocation of fire protection, fire prevention, and plant restoration resources to areas with the highest fire risk. The characteristics used for forest fires, such as soil type, topography, humidity, and rainfall, are very different from those typically used in urban fire assessment and prediction (e.g., combustibility of building materials, fire performance, and type of property use).

### 2.1. Application and Research Status of Forest Fire

Meriame [14] developed five new hybrid machine learning algorithms combining frequency ratios with multilayer perceptron, logistic regression, categorical regression tree, support vector machine, and random forest for mapping deep forest fire susceptibility in northern Morocco. Tuyen [15] proposed four new integrated models combining locally weighted learning (LWL) algorithms with cascaded generalization (CG), bagging, decorate, and dagging integrated learning techniques to predict fire sensitivity in a spatially explicit manner. The results show that the CG-LWL and bagging-LWL models with AUC = 0.993 are the best trained. Integrating models to improve the predictive accuracy of forest fire sensitivity can save time and costs in firefighting efforts. However, despite the good performance and obvious advantages of these excellent models for forest fire prediction, they are not applicable to building fire prediction or stadium fire prediction studies.

### 2.2. Application and Research Status of Urban Building Fire

Static risk assessment. Wei [16] proposed a rapid fire assessment method based on fuzzy mathematics and support vector machines to obtain index values as well as sample data for fire risk scoring by fuzzy comprehensive evaluation, and a support vector machine fire risk assessment model trained on the sample data was used for stadium fire risk assessment to verify the feasibility of the assessment. Lau [17] assessed the fire risk of each building and determined its risk level by determining the weights of individual indicators through hierarchical analysis, and the results were validated using a support vector machine model. This method is a deterministic assessment method with a complex mathematical model, high risk assessment cost, long calculation time consumption, and inability to realize dynamic assessment, which is mainly applicable to the fire risk assessment needs of special buildings and difficult to be promoted on a large scale.

Dynamic risk assessment. Some scholars have focused on “dynamic” needs. Liu [18] proposed a cross-regional transfer learning approach to identify fire hazards framework in communities (e.g., parking lots, public spaces, and shopping centers). The recognition performance was improved by classifying community fire hazards into nine classes, and the overall accuracy, accuracy, recall, F1 score, and AUC were improved by 12%, 15%, 16%, 15%, and 15%, respectively. Firebird [19] is a model for predicting building fire risk in Atlanta. It uses fire event data (time, location, and cause of fire), commercial property structure data, property fire risk inspection data, and predicted fire risk scores between 0 and 1 for the building industry. The results were evaluated as the best performance of the random forest (RF) model with an AUC value of 0.8246.

## 3. Methodology

### 3.1. Data Collection and Preprocessing

Due to various reasons such as failure of IoT sensing devices (e.g., intermittent loss of sensor connections), human negligence, and technical problems with IoT remote monitoring systems and cloud servers, data collection conditions are not perfect and may result in noisy data containing missing, redundant, and erroneous data. Obviously, information extracted from noisy data (i.e., unreliable data) can be wrong and therefore lead to day-to-day management decisions that are likely to be irrational. In addition, the unit measures of various fire risk hazard characteristics in the collected data are not uniform, which is not conducive to the construction of classification prediction models. Therefore, all the above issues must be addressed in the preprocessing stage by applying various preprocessing operations such as data cleaning, data transformation, and other data enhancement methods.

In order to quantify and grade the prediction results, we designed quantifiable threshold intervals and thresholds to quantify and grade the indicators (data conversion). Specifically, Table 1 demonstrates an example of equipment management fire risk indicators. If the collected data are missing more than 30% of the set threshold, they are complemented using the interpolation method (data cleaning). For discrete features, such as fire host failure ratio, the mean interpolation method is used to complement the median of the features. For category features, such as spray control cabinet status, smoke control cabinet status, and other indicators, they are quantified according to design thresholds and supplemented with discrete feature types. The mean interpolation and plural interpolation are mainly used to fill in the rest of the data predictions based on this feature to eliminate noise and correct inconsistencies.

In this paper, we collected 176 stadium fire risk datasets and 289 equipment management datasets. For the experiments, the datasets we used are divided into two folders: training set (70%) and testing set (30%) (See Table 2), which are used to train and validate the validity and accuracy of the models. Meanwhile, Appendix A and Appendix B present the data types and values of the two data sets mentioned above.

### 3.2. Comparison and Selection of Machine Learning Algorithms

Machine learning algorithms are not one-size-fits-all, and no scientific validation is given as to which algorithm is more suitable for stadium fire risk prediction; multidimensional experimental studies on multiple classification algorithms and model testing methods are needed. According to Table 3, it can be seen that the random forest algorithm outperforms other machine learning methods in every performance index, so random forest is chosen as the experimental model in this paper. To further improve the performance of the model, we fine-tune and optimize the model parameters in order to configure the simulation environment parameters that are most suitable for the stadium fire risk dataset. The optimal simulation environment parameter setting is n_estimators = 2000, min_samples_split = 5, min_samples_leaf = 1, max_features = sqrt, max_depth = 10, bootstrap = True.

### 3.3. SHapley Additive exPlanations (SHAP) Approach

SHAP is based on an approach subject to cooperative game theory [20], which centers on the introduction of Shapley values to construct an additive explanatory model that facilitates the interpretation of the performance of machine learning models or deep learning models, i.e., Shapley values [21] measure the marginal contribution of each input variable in the overall cooperation. This approach falls under the category of additive feature attribution, and a surprising property in this category is the existence of a single unique solution that has three desirable properties: local accuracy, missingness, and consistency.

**Figure 1:**
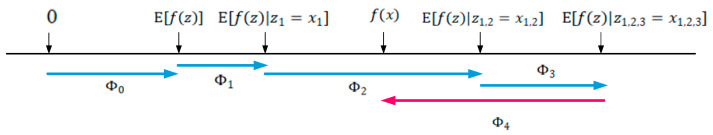
SHAP attributes.

Property 1 (local accuracy): When approximating the original model *f* for a particular input *x* local accuracy [22] requires that the explanatory model g at least match the output of *f* for the simplified input $\;x^{\prime }$. Equation (1) is explained in Figure 1, where $\phi_{0}$, $\phi_{1}$, $\phi_{2}$, and $\phi_{3}$ increase the predicted value of *g()*, while $\phi_{4}$ decreases the predicted value of *g()*.

(1) $$f\left(x\right)=g\left(x^{\prime }\right)=\phi_{0}+\overset{M}{\underset{i=1}{\sum }}\phi_{i}x_{i}^{\prime }$$

The explanation model $g\left(x^{\prime }\right)$ matches the original model f(x) when $x=h_{x}\left(x^{\prime }\right)$, where $\phi_{0}$ = $f\left(h_{x}\left(0\right)\right)$ represents the model output with all simplified inputs toggled off.

Property 2 (missingness): The missing features in the original input are not important.

(2) $$x_{i}^{\prime }=0\Rightarrow \phi_{i}=0$$

Missingness constrains features where $x_{i}^{\prime }=0$ to have no attributed impact.

Property 3 (consistency): Consistency means that even if we change a model so that a feature has a greater impact on the model, the attributes assigned to that feature are not reduced [22].

(3) $$F_{x}^{\prime }\left(z^{\prime }\right)-f_{x}^{\prime }\left(z^{\prime }\backslash i\right)\geq f_{x}\left(z^{\prime }\right)-f_{x}\left(z^{\prime }\backslash i\right)$$

for all inputs *z′*∈{0,1}, then $\phi_{i}$*(f′, x) ≥* $\phi_{i}$*(f, x).*

One drawback of the above elaborated properties 1, 2, and 3 is that other additive feature attribution methods are unknown [23], although for estimating Shapley worth, classical methods are familiar and knowable. Therefore, we introduce other methods to solve this nuisance, and the only possible model for Equation (4) to satisfy these properties is

(4) $$\phi_{i}\left(f,x\right)={\sum^{\;}}_{z^{\prime }\subseteq x^{\prime }}\frac{\left|z^{\prime }\right|!\left(M-\left|z^{\prime }\right|-1\right)!}{M!}\left[f_{x}\left(z^{\prime }\right)-f_{x}\left(z^{\prime }\backslash i\right)\right]$$

where *|*$z^{\prime }$*|* is the number of nonzero entries in $z^{\prime }$, and $z^{\prime }$ ⊆ $x^{\prime }$ represents all $z^{\prime }$ vectors where the nonzero entries are a subset of the nonzero entries in $\;x^{\prime }$. Lundberg and Lee [24] suggested a solution to Equation (4) where $f_{x}\left(z^{\prime }\right)=f\left(h_{x}\left(z^{\prime }\right)\right)=E\left[f\left(z\right)|z_{S}\right]$ and *S* is the set of nonzero indices in *z’*, known as SHAP values.

Contrary to the existing interpretation of significant features in machine learning models, SHAP has the advantage of identifying whether the contribution of each input feature is positive or negative. In addition, SHAP can construct explanatory models that provide good explanations for both local and global models. SHAP values can be approximated by various methods, such as Deep SHAP, Kernel SHAP, and Tree SHAP. This study uses the Tree SHAP method, which is a tree-based machine learning model such as random forest (RF), decision tree, and gradient boosted tree (AdaBoost, CatBoost, LightGBM and XGBoost).

### 3.4. Approach for Interpretable Machine Learning

**Figure 2:**
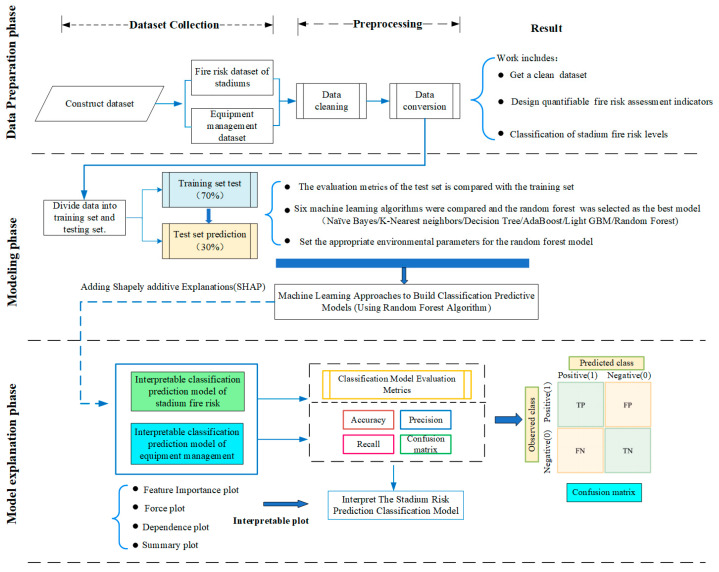
The key process using SHAP for an interpretable machine learning model.

To ensure the quality of the experimental results, in this study we propose an interpretable machine learning model architecture that consists of three phases. Figure 2 illustrates the methodology for identifying the important variables of the stadium fire risk assessment model and the equipment management risk assessment model. In the data preprocessing phase, the quality of the dataset is assessed based on the percentage of missing values and it is preprocessed to become a clean dataset (data cleaning). Then, because the unit measures of various fire risk hazard characteristics are not uniform, quantifiable threshold intervals are designed to classify and quantify various fire risk factors (data conversion). In the modeling phase, an experimental comparison study was conducted with six machine learning algorithms, using different metrics to measure the performance of the models, and the best performing model was decided. Finally, a random forest model with SHAP strategy is combined in the assessment phase in order to build a stadium fire risk assessment model. The impact analysis of each risk factor on different risk assessment models is analyzed according to interpretable plots (including feature important plot, force plot, dependence plot and summary plot). Section 3.1 to Section 3.3 describe in more detail the data preprocessing, the comparison and selection of machine learning models, and the introduction of SHapley Additive exPlanations strategy to build a stadium fire risk prediction model.

## 4. Experimental Results and Discussion

### 4.1. Experimental Environment

We used a computer to implement and test the configuration of our proposed idea. This computer is a personal computer using the Windows operating system, as shown in Table 4.

### 4.2. Identification of Importance Factors for Fire Risk Assessment Modes of Stadiums

The experimental database of stadium fire risk consists of 176 samples, of which 29, 68, 43, and 36 samples are distributed in Ideal Safety mode, Safety mode, Critical mode, and Hazardous mode (Table 5 presents the classification of stadium fire risk assessment modes). Based on the available statistics of causal factors [25], the assessment model of stadium fire risk was predicted using five input variables: building inherent safety (BIS), safety personnel management (SPM), fire protection base data (FPBD), equipment management (EM), and hidden danger management (HDM).

**Figure 3:**
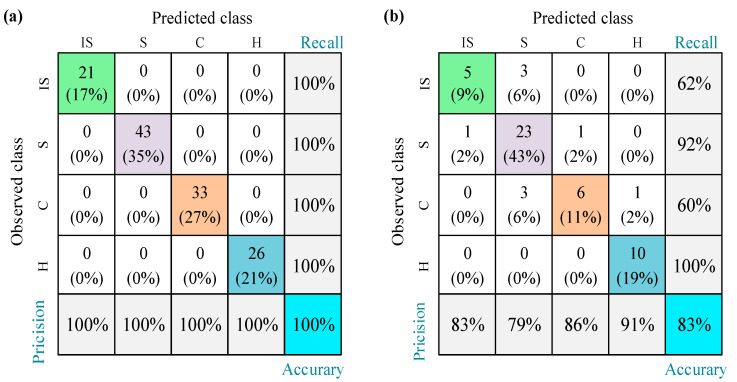
Confusion matrix of an RF classifier model for the fire risk database of stadium: (**a**) training set and (**b**) test set (IS: Ideal Safety, S: Safety, C: Critical, and H: Hazardous).

As shown in Figure 3, the accuracy of the RF model selected in this study is 100% and 83% for the training and test sets, respectively. Accuracy is the fraction of samples correctly predicted by the classifier. Note that the sensitivity of the RF model to the training and test sets was not evaluated in the current study, and the selection of the best machine learning model for the stadium fire risk assessment model and the equipment management risk assessment model was not discussed. The scope of our study is limited to the interpretation of machine learning models using SHAP. The performance of the model is analyzed by means of a confusion matrix that displays a table of observed versus predicted risk assessment modes. In the table, the diagonal elements indicate the predicted correct evaluation model. Other performance metrics evaluated by the model are precision and recall. The percentage of predicted risk assessment modes correctly classified by the model is the accuracy (fifth row of the confusion matrix, Figure 3). The actual risk assessment model correctly established by the machine learning model is recall. As shown in Figure 3, the model has high accuracy and recall in identifying Hazardous mode.

**Figure 4:**
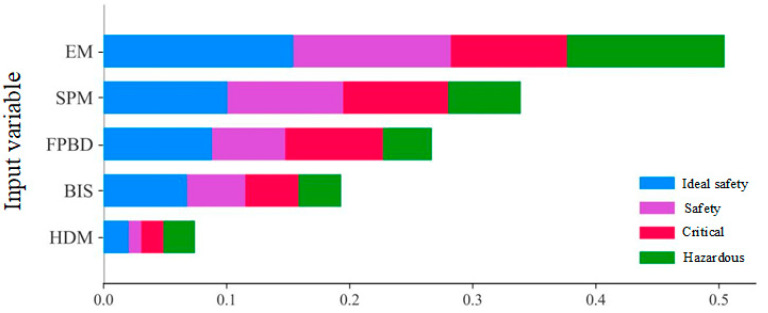
Importance factor of each input variable for fire risk database of stadiums.

The effect of input variables on the prediction of RF models for stadium fire risk assessment models can be further explored by SHAP. The global importance factors of the five input variables are shown in Figure 4. The average of the absolute Shapley values for each feature in the global importance estimation data is shown. The input variables are ranked by importance, that is, the higher the average SHAP value, the more important the variables are. In addition, Figure 4 shows the importance of each input variable for the four risk assessment modes of Ideal Safety mode, Safety mode, Critical mode, and Hazardous mode; thus, the figure provides additional insight into the prediction of fire risk assessment modes for stadiums that have not yet been explored. The current study has not only global outputs but also individual categories (Ideal Safety, Safety, Critical, Hazardous). According to the global output, hidden danger management (HDM) is the least important and equipment management (EM) is the most important.

**Figure 5:**
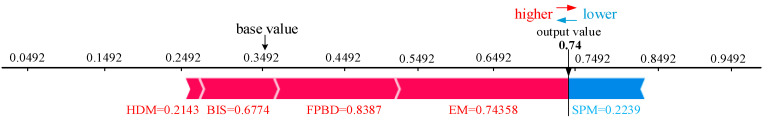
Explanation of the Safety mode.

Figure 5 shows the prediction plots of the input variables for Safety mode. The SHAP value enables the decomposition of the Safety mode prediction into the sum of the effects of each input variable. The predicted and observed assessment mode is Safety mode. Figure 5 shows the actual contribution of these factors to Safety mode. In Figure 5, the blue arrows indicate the variables that affect the predictions of the other evaluation modes, and the red arrows indicate the variables that push the predictions to Safety mode. The base value is the proportion of samples that belong to a specific category. For example, the base value in Figure 5 corresponds to the proportion of samples in Safety mode in the overall sample (i.e., 68/176). In other words, the base value is the probability of predicting Safety mode when there is no information about the input variables. Safety mode predictions with probability values higher than 0.3492 may lead to a tendency to shift to assessment modes with a higher probability of fire risk occurrence (e.g., Hazardous mode). However, the predicted probability of Safety mode is 0.74, which indicates a high probability of Safety mode, i.e., the stadium is in a stable and safe condition. In Figure 5, the Safety mode prediction is contributed by the values EM and FPBD. The variable FPBD (SHAP value of 0.8387) is the most important factor that pushes the RF machine learning model prediction to predict Safety mode.

**Figure 6:**
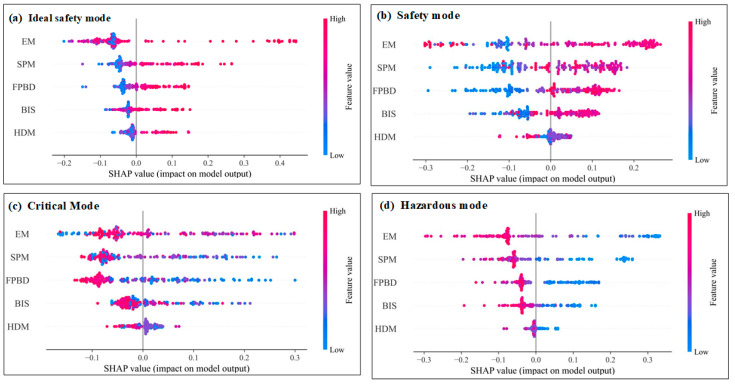
Summary plots for various assessment mode of fire risk.

The range and distribution of the influence of the input variables on the stadium fire risk assessment model can be shown by summary plots (Figure 6). Each point on the fluctuation graph in Figure 6 is a Shapley value for the input variable and an instance. The *y*-axis is sorted by the input variables from top to bottom in importance, and each point is colored by the value of the input variable, from low (blue) to high (red). The position on the *x*-axis is determined by the Shapley value. Overlap points represent the distribution of points in the dataset, i.e., it indicates the range contained in the values. Figure 6a–d represent Ideal Safety mode, Safety mode, Critical mode, and Hazardous mode, respectively, where equipment management (EM) is the most important factor in determining the fire risk assessment of stadiums. Figure 6b shows that the higher the EM value is, the larger its SHAP value is and the greater its effect on Safety mode. SPM, FPBD, and BIS are the next key factors, and an increase in the value of all three leads to an increase in Safety mode potential. However, although hidden hazard management (HDM) is the least significant, it tends to reduce the possibility of safety mode as its value increases. On the other hand, low values of EM, SPM, and FPBD increased the probability of correlation between SHAP and predicted Critical mode as well as Hazardous mode (Figure 6c,d). For Critical mode or Hazardous mode, low values of EM, SPM, FPBD, and BIS tend to increase the probability of Critical mode or Hazardous mode (convergence toward the risky unstable state). However, similar to Safety mode, a higher HDM value reduces the likelihood of Critical mode and Hazardous mode. Furthermore, Figure 6a shows that high values of EM, SPM, FPBD, and BIS tend to increase the likelihood of Ideal Safety mode, which is the same as Safety mode. Note that, in contrast to Safety mode, high values of HDM tend to increase the likelihood of Ideal Safety mode. As shown in Figure 6a,b, the variation of HDM determines that the assessment mode is Ideal Safety mode, Safety mode. This variable has a significant difference on Ideal Safety mode, Safety mode. It is important to note that it is often difficult to identify Critical mode and Hazardous mode, and an extensive database is needed to determine the decision boundaries between Critical mode and Hazardous mode and with other assessment modes. The insights in Figure 6 help domain experts plan experimental studies and help establish Critical mode and Hazardous mode boundaries or closed form solutions.

**Figure 7:**
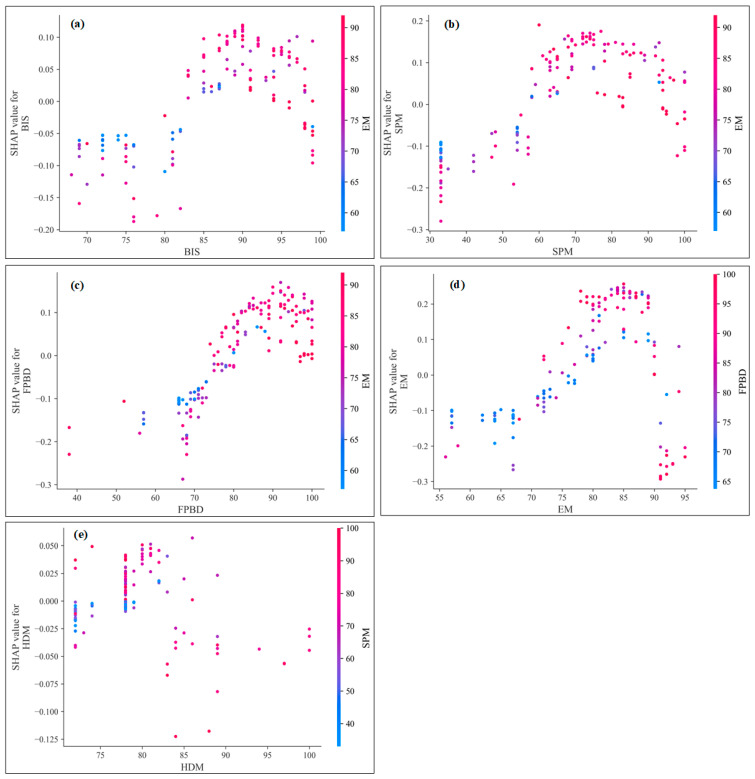
SHAP dependency plots for Safety mode of the fire risk database of stadiums.

Figure 7 shows the SHAP dependence of Safety mode, where the SHAP value varies with the input variables. Although the SHAP values shown in Figure 6 and Figure 7 are the same, Figure 7 shows the marginal effects of one or both input variables on the predicted outcomes of the RF machine learning model and can show whether the relationship between the risk assessment model and the input variables is linear, monotonic, or more complex. In Figure 7b, the effect of SPM is shown as the EM is varied from 60 to 90. Red values indicate high values of the variable EM, while blue indicates low values. When the SPM is higher than 60, the SHAP value of EM is positive. For the high values of SPM and EM, the SHAP values are extremely high. That is, high values of SPM and EM lead to a higher probability of Safety mode. For EM values above 70 and SPM greater than 60, there is a clear trend of EM and SPM on Safety mode prediction.

### 4.3. Identification of Importance Factors for Equipment Management Assessment Modes

In Section 4.2 we describe that equipment management (EM) is the most important factor to measure the fire risk of stadiums and has a tendency to contribute to their increased fire risk. In order to further investigate the variables affecting stadium fire risk, we will therefore explore the influence of the input variables of equipment management on them, thus indirectly explaining the relationship between the input variables of equipment management on stadium fire risk. An experimental database consisting of 130 ideal safety models, 72 safety models, 54 critical models, and 33 hazard models was used in this study.

**Figure 8:**
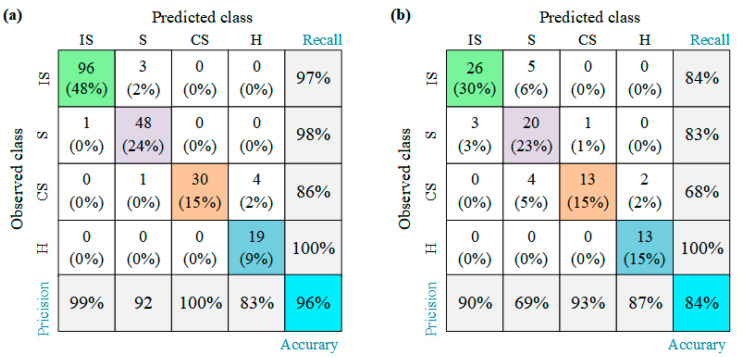
Confusion matrix of an RF classifier model for the equipment management database: (**a**) training set and (**b**) test set.

**Figure 9:**
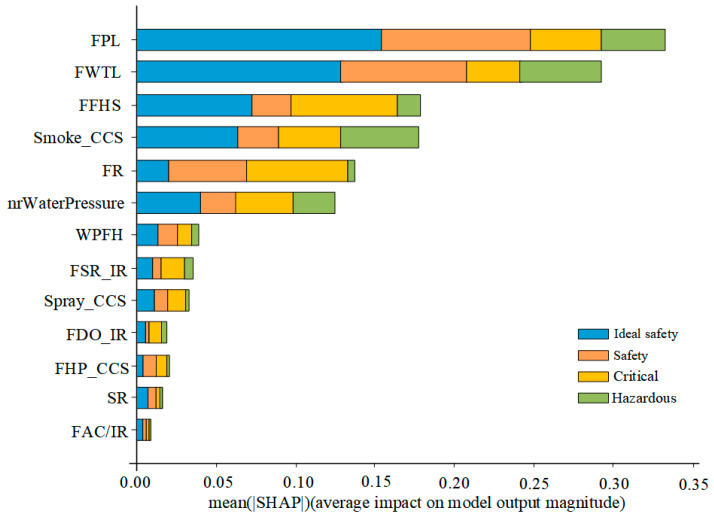
Importance factor of each input variable for the equipment management database.

As shown in Figure 8, the RF model has an accuracy of 96% for the training set and 84% for the test set. Figure 9 shows the important factors that identify the input variables when the equipment management has the various evaluation modes. FPL is the most important factor in equipment management, followed by FWTL, FFHS, and Smoke_CCS. In addition, Figure 9 also shows that the importance of the input variables varies according to the different risk assessment models.

**Figure 10:**
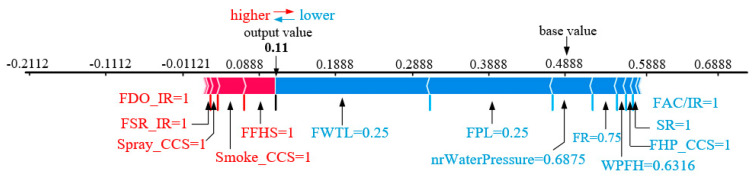
Explanation of the Ideal Safety mode.

Figure 10 shows the prediction plots of the input variables for Ideal Safety mode. Although the baseline value is 0.4888, the RF model predicts an Ideal Safety mode probability of 0.11 (lower than the baseline value). The individual contributions of each input variable are also shown in Figure 10, where FFHS = 1, Smoke_CCS = 1, Spray_CCS = 1, FSR_IR = 1, and FDO_IR = 1 are the factors driving the value up.

**Figure 11:**
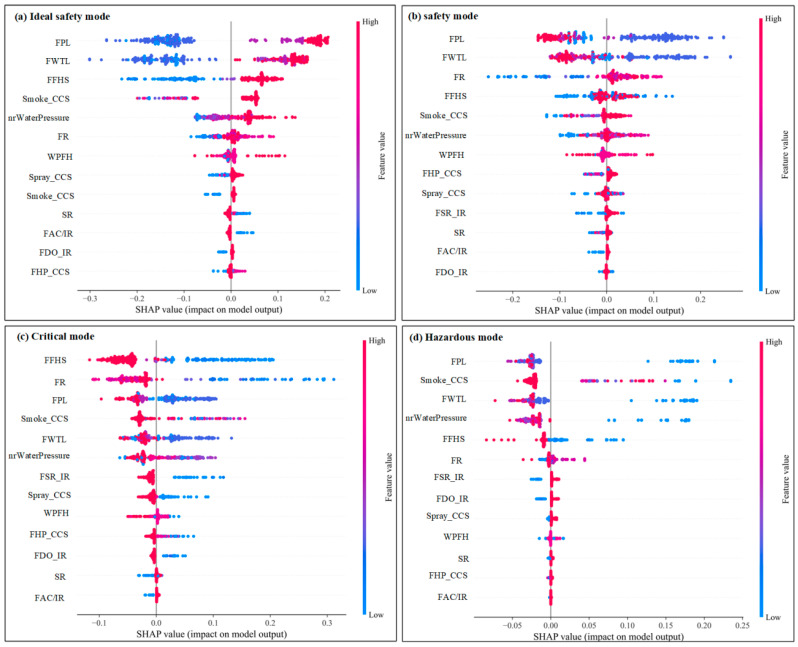
Summary plots for various mode of equipment management database.

Figure 11 shows the effect of the distribution of input variables on the various risk assessment models. The results show that an increase in the fire pool level (FPL) leads to higher SHAP values and their associated probabilities for the Ideal Safety mode. The variable FPL has the most significant effect on the risk assessment model beyond the critical model. Figure 11 also shows the importance of various input variables for different risk assessment models. FFHS is most important for the critical mode, and high values of FFHS reduce the sensitivity of the critical mode. FDO/IR and FAC/IR have less impact on Ideal Safety mode and Safety mode. FHP_CCS, SR, and FAC/IR have less impact on the Hazardous mode. As expected, Smoke_CCS severely affects the Hazardous mode.

**Figure 12:**
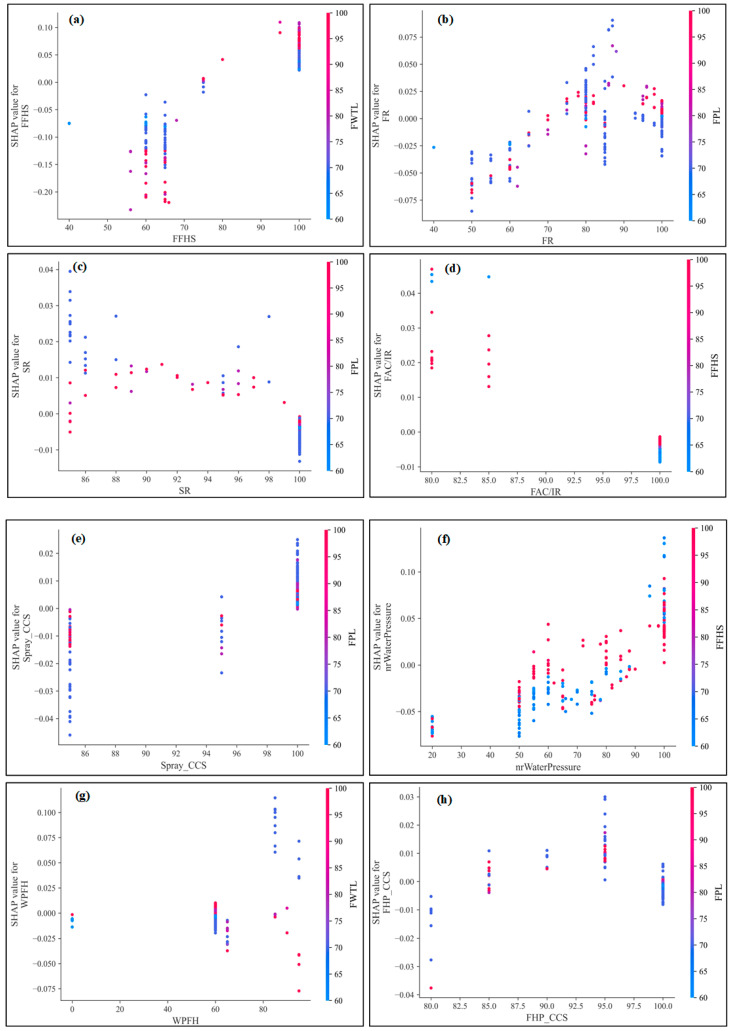

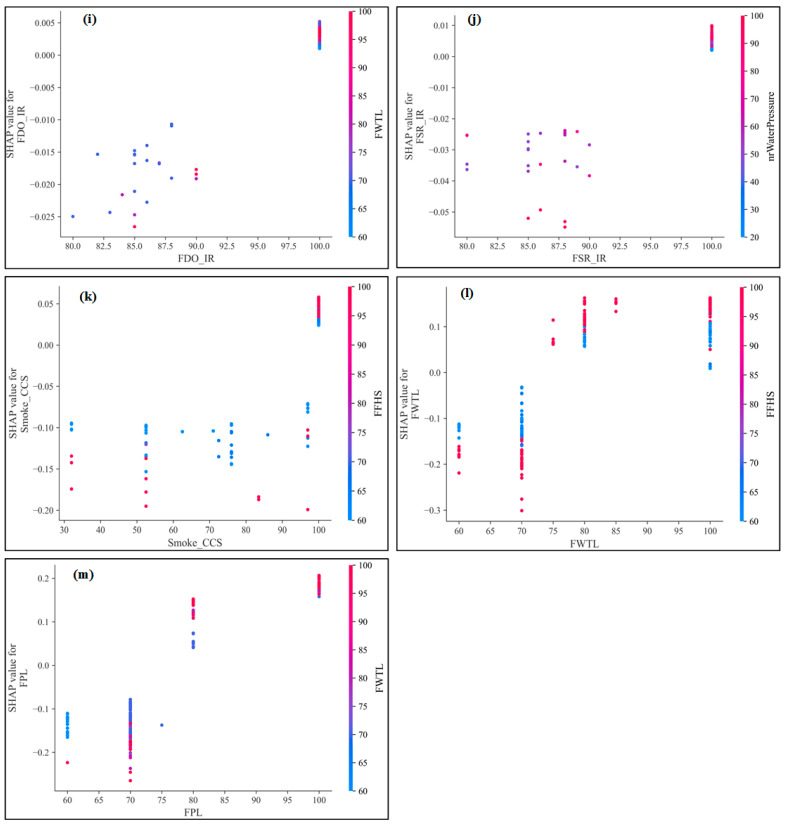
SHAP dependency plots for Ideal Safety mode of the equipment management database.

Figure 12 shows the SHAP dependence diagram of the Ideal Safety model of the equipment management database as a function of various input variables estimated using the machine learning model. Ignoring the colors in Figure 12a, SHAP is positive when the FFHS value is greater than 70, which indicates that the probability of the Ideal Safety mode increases as the FFHS value exceeds 70. Additionally, the associated SHAP values and Ideal Safety mode probabilities increase when the FR is greater than 70 and the FPL is greater than 80 (Figure 12b). As shown in Figure 12c,d, there is no clear pattern in SHAP values as SR and FAC/IR change. Similar conclusions apply to nrWaterPressure, WPFH, and Smoke_CCS.

### 4.4. Comparison with Other Study

This section shows the performance of the proposed model compared with studies related to fire risk identification and prediction using machine learning or deep learning methods. Table 6 shows the performance tests of the model proposed in this study as well as the results obtained from existing studies. Refs. [26,27] deep learning approach (Detectron2) and a new special convolutional neural network (Improved YOLOv3) to build detection platforms or systems for high accuracy and fast detection and recognition of forest fire image recognition (both day and night), respectively. The scope of this paper refers to fire risk assessment and prediction, and does not include image recognition and detection techniques. However, since the use of machine vision images acquired by fire detection cameras as input features is a trend and background for our future research, and since Detectron2 and Improved YOLOv3 methods have achieved good model performance in fire detection and recognition, they can be used as desired implementations of the concept. In addition to the two mentioned above, this performance test shows that the proposed model outperforms existing fire risk assessment and prediction studies, with our top model performing at 83% accuracy, 85% recall, and 86% precision. Therefore, the proposed prediction model in this study is acceptable in terms of risk prediction.

## 5. Limitations

This study also encountered some limitations. First, there is scarcity in the dataset. Although the data are of low order of magnitude and barely meet the minimum requirements of the experimental model, the amount of data in Critical mode and Hazardous mode is still scarce. Therefore, we need to collect an extensive database that will help domain experts to plan experimental studies to determine decision boundaries or establish closed form solutions for Critical mode and Hazardous mode and other assessment modes (Section 4.3). Second, the dataset is relatively homogeneous. The fusion of a large amount of other types of data (e.g., fire images, time series data) may improve the performance of the model. In the experimental study, we only considered one response variable, stadium fire risk, which obviously affected the usefulness of the model. The application value of the method would be greater if more accurate data on property damage, casualties, social impact, and other indicators could be integrated into the prediction model. In addition, the data categories are unbalanced. The experimental dataset used in this paper did not consider the problem of category imbalance, and we should explore ways to solve the label imbalance, while paying attention to overfitting and underfitting, to maximize the validity and accuracy of the model. Therefore, potential improvements need to be further investigated and planned for in our future work.

In future work, as researchers acquire an overwhelming amount of data, a single machine learning approach can no longer handle it effectively. Therefore, we need some measures to reduce operational efficiency and time cost. Ensemble learning is very popular in recent research on fire risk assessment and prediction. In contrast to a single machine learning approach, it is an integration of several different weak classifiers to form a strong classifier. The classification result is decided jointly by multiple classifiers voting to reduce the impact of individual classifier errors thus improving classification accuracy and stability. For example, Zhang [33] proposed a recurrent LSTM neural network (R-LSTM-NN) for predicting fire hazard values in smart cities, and the proposed model detected fire outbreaks with 98.4% accuracy and a minimum error rate of 0.14%.Ying [34] proposed a forest fire prediction model based on integrated extreme gradient boosting algorithm and random forest algorithm to predict the frequency of forest fires and fire burning area. Prediction performance better than that of single learning methods such as support vector machines, random forests, artificial neural networks, deep learning, decision trees, and extreme gradient boosting was obtained, demonstrating the superiority and high generalization capability of the algorithm, which provides an important technique for forest firefighting decisions in terms of fire resource allocation and strategies.

## 6. Conclusions

In this study, relying on the IoT remote fire monitoring system platform to collect IoT monitoring data features, we are committed to designing a quantifiable threshold interval to quantify and grade the indicators of different data types to obtain two quantified datasets. In the experiments, the data samples were divided into two folders: training set (70%) and test set (30%) to construct interpretable machine learning models. We combined the random forest model of SHAP strategy to establish the stadium fire risk assessment model and analyzed the impact analysis of each risk feature on four different risk assessment models, so as to find the complex nonlinear relationship between risk features and stadium fire risk. The experimental study showed that for stadium fire risk, the developed interpretable random forest model provided 83% accuracy, 86% precision, and 85% recall for the stadium fire risk test dataset. In addition, EM was the key factor in determining the risk assessment model, followed by SPM, FPBD, and BIS. An increase in EM leads to an increase in the likelihood of Safety mode, while an increase in the values of SPM, FPBD, and BIS will increase the Safety mode trend. Hidden danger management (HDM) is the least important, but an increase in its value decreases the likelihood of Safety mode. The experimental results also show that the low data magnitude leads to difficulties in identifying the decision boundary ranges of Critical mode and Hazardous mode. Finally, a cloud platform based on Chinese IoT Big Data to obtain a broader sense of stadium fire data is applied for future research of this machine learning approach.

## Figures and Tables

**Table 1:** Examples of fire risk indicators for equipment management.

Response Variable	Input Features	Level I [90–100]	Level II [80–90)	Level III [70–80)	Level IV [60–70)	Level V (<60)
Equipment management	Fire host status	Normal	—	No data	Offline time ≤ 24 h	Offline time > 24 h
	Spray control cabinet status	Automatic	Manual	Offline	—	Disconnected
	Integrity rate of fire alarm controller	100%	[95%, 100%)	[90%, 95%)	[80%, 90%)	<80%
	Failure ratio	0%	(0%, 5%]	(5%, 10%]	(10%, 20%]	>20%
	Shielding ratio	0%	(0%, 5%]	(5%, 10%]	(10%, 20%]	>20%
	Smoke control cabinet status	Automatic	Manual	Offline	—	Disconnected
	Worst point fire hydrant water pressure	≥0.05 MPa			<0.05 MPa	
	Fire water tank level	[0, 50 mm)	[50 mm, 100 mm)	>100 mm		
	Fire pool level	[0, 50 mm)	[50 mm, 100 mm)	>100 mm		

**Table 2:** Distribution of stadium fire risk data and equipment management data.

Dataset	Training Set	Testing Set	Total
Stadium fire risk	123	53	176
Equipment management	202	87	289

**Table 3:** Using stadium fire risk datasets for model fitting and selection of optimal models.

Machine Learning Algorithms	Weighted Performance Metrics			
	Accuracy	Precision	Recall	F1-Score
Naive Bayes	0.49	0.65	0.49	0.46
K-nearest neighbors	0.75	0.74	0.75	0.74
Decision tree	0.74	0.74	0.74	0.73
AdaBoost	0.62	0.61	0.62	0.58
Light GBM	0.81	0.81	0.80	0.80
Random forest	0.83	0.86	0.85	0.82

**Table 4:** Windows desktop specifications used for the experiments.

Hardware	Detailed Specifications
Processor	Intel Core i7-10750H 260 GHz
GPU	NVIDIA Quadro T2000,
Memory	16 GB DDR4
Motherboard	SDK0L77769 WIN
Storage	1024 GB M.2, 4 TB Hard Drive
Operating system	Windows 12 Pro
Power	LGC 5B10W13958

**Table 5:** Classification of fire risk assessment modes of stadiums.

Risk Assessment Mode	Risk Value	Attribute Requirements		
		Hidden Dangers Frequency	Fire Frequency	Casualties/Property Losses
Ideal Safety mode	[90–100]	Extremely low	Extremely low	No/No
Safety mode	[80–90)	Low	Low	No/Minor
Critical mode	[70–80)	Medium	Medium	Partial/Large
Hazardous mode	[60–70)	High	High	Partial/Major

**Table 6:** Comparison of the performance achieved by the proposed model with existing research.

Algorithm	Accuracy	Recall	F1-Score	Precision
Detectron2 [26]	—	99.4%	95.5%	99.3%
Improved YOLOv3 [27]	—	99.2%	99.5%	98.1%
Deep neural network [28]	75.1%	—	—	—
SVM [29]	78.0%	—	—	—
AdaBoost [30]	71.0%	—	69.0%	—
Logistic regression [31]	—	80.3%	78.3%	—
Neural networks [32]	72.5%	55.8%	40.0%	76.3%
Our method (random forest)	83.0%	85.0%	82.0%	86.0%

## Data Availability

Not applicable.

## Appendix A

**Table A1:** Description of attributes from Stadium Fire Risk Dataset.

Attribute Name	Description	Data Type and Value
Fire risk of stadiums:		Nominal-Ideal safety mode, Safety mode, Critical mode, Hazardous mode
BIS	Building inherent safety	Numerical
SPM	Safety personnel management	Numerical
FPBD	Fire protection base data	Numerical
EM	Equipment management	Numerical
HDM	Hidden danger management	Numerical

## Appendix B

**Table A2:** Description of attributes from equipment management database.

Attribute Name	Description	Data Type and Value
Equipment management:		Nominal-Ideal safety mode, Safety mode, Critical mode, Hazardous mode
FFHS	Fire fighting host status	Nominal-Normal, no data, offline duration (≤24 h), offline duration (>24 h)
FR	Fire host failure ratio	Numerical-%
SR	Fire host shielding ratio	Numerical-%
FAC/IR	Integrity rate of fire alarm controller	Numerical-%
Spray_CCS	Spray control cabinet status	Nominal-Automatic/ manual/offline/disconnected
nrWaterPressure	Normal rate of water pressure at the end of sprinkler system	Numerical-%
WPFH	Worst point fire hydrant water pressure	Numerical- MPa
FHP_CCS	Fire hydrant pump control cabinet status	Nominal-Automatic/ manual/offline/disconnected
FDO_IR	Fire door operating integrity ratio	Numerical-%
FSR_IR	Fire shutter running integrity ratio	Numerical-%
Smoke_CCS	Smoke control cabinet status	Nominal-Automatic/ manual/offline/disconnected
FWTL	Fire water tank level	Numerical- mm
FPL	Fire pool level	Numerical- mm
